# Physical activity and screen time in children and adolescents in a medium size town in the South of Brazil

**DOI:** 10.1016/j.rppede.2016.01.001

**Published:** 2016

**Authors:** João Paulo de Aguiar Greca, Diego Augusto Santos Silva, Mathias Roberto Loch

**Affiliations:** a Brunel University London, Uxbridge, Greater London, United Kingdom; b Universidade Federal de Santa Catarina (UFSC), Florianópolis, SC, Brazil; c Universidade Estadual de Londrina (UEL), Londrina, PR, Brazil

**Keywords:** Sedentary lifestyles, Socioeconomic factors, Leisure activities, Television, Obesity

## Abstract

**Objective:**

To analyze the associations between sex and age with behaviour related to physical activity practice and sedentary behaviour in children and adolescents.

**Methods:**

A cross-sectional study with 480 (236 boys) subjects enrolled in a public school in the city of Londrina, in the south of Brazil, aged 8–17 years. Measures of physical activity, sports practice and screen times were obtained using the Physical Activity Questionnaire for Older Children. The Mann–Whitney *U* test was used to compare variables between boys and girls. The Chi squared test was used for categorical analysis and Poisson regression was used to identify prevalence.

**Results:**

Girls (69.6%; PR=1.05 [0.99–1.12]) spent more time with sedentary behaviour than boys (62.2%). Boys (80%; PR=0.95 [0.92–0.98]) were more physically active than girls (91%). Older students aged 13–17 showed a higher prevalence of physical inactivity (91.4%; PR=1.06 [1.02–1.10]) and time spent with sedentary behaviour of ≥2h/day (71.8%; PR=0.91 [0.85–0.97]) when compared to younger peers aged 8–12 (78.7 and 58.5%, respectively).

**Conclusions:**

The prevalence of physical inactivity was higher in girls. Older students spent more screen time in comparison to younger students.

## Resumo

**Objetivo:**

Analisar a associação do sexo e idade com comportamentos relacionados à prática de atividades físicas e sedentarismo em crianças e adolescentes.

**Métodos:**

Estudo transversal com 480 (236 sexo masculino) estudantes de uma escola pública de Londrina, Paraná, Brasil, entre 8 e 17 anos. As medidas de atividade física, prática de esportes e quantidade de comportamentos sedentários foram obtidas mediante aplicação do *Physical Activity Questionnaire for Older Children*. O teste U de Mann-Whitney foi usado para comparar variáveis de rapazes e moças. O teste de qui-quadrado foi usado para variáveis categóricas e a regressão de Poisson para identificar prevalências.

**Resultados:**

Moças (69,6%; RP=1,05 [0,99-1,12]) dedicaram mais tempo ao comportamento sedentário quando comparadas com rapazes (62,2%). Rapazes (80%; RP=0,95 [0,92-0,98]) apresentaram maiores níveis de atividade física quando comparados com moças (91%). Estudantes mais velhos entre 13-17 anos (91,4%; RP=1,06 [1,02-1,10]) apresentaram maior prevalência de inatividade física e comportamento sedentário de ≥2h/dia (71,8%; RP=0,91 [0,85-0,97]) quando comparados com estudantes entre 8 e 12 anos (78,7 e 58,5%, respectivamente).

**Conclusões:**

A prevalência de inatividade física foi superior entre as moças. Estudantes mais velhos despenderam mais tempo em tela quando comparados com estudantes mais novos.

## Introduction

The current literature reports that higher levels of physical activity can reduce the risk of premature all-cause mortality, and also supports the dose-response relationship between physical inactivity and chronic conditions, *i.e.* cardiovascular disease, stroke, hypertension, colon cancer, breast cancer, type 2 diabetes and osteoporosis.^1^ Studies have shown that increased sedentary behaviours, such as television viewing, video game playing, playing computer games, and/or electronic game playing, are associated with unfavourable body composition, decreased fitness, lowered scores for self-esteem and pro-social behaviour and decreased academic achievement in school-aged children.^2^

Low levels of physical activity in childhood and adolescence have been reported worldwide, with a proportion of 80.3% doing fewer than 60min of physical activity of moderate to vigorous intensity per day.^3^ A study describing adolescents’ physical activity levels with data from 32 countries concluded that the majority of adolescents do not meet current recommendations of physical activity.^4^ In Brazil, high levels of physical inactivity in children and adolescents were reported in the southern^5^ and northeast regions.^6^

Sedentary behaviour is related to an unhealthy lifestyle early in childhood and adolescence. Watching television for more than two hours, for instance, increases the chances of overweight and obesity as reductions in sedentary behaviour are linked to better body composition.^2^ Recent publications have shown that sedentary behaviour in young people, especially in the form of TV viewing, is associated with a less healthful diet, such as less fruit and vegetable consumption and a greater consumption of energy-dense snacks and beverages containing sugar.^7^^,^ 8 Moreover, behaviours established in school-age children tend to continue into adulthood^9^ and studies that include this population have been suggested.^1^

Some previous Brazilian studies involving physical inactivity and sedentary behaviour focused on investigating adolescents^5^^,^ 6 but did not stratify subgroups *i.e.* age and gender comparisons as recommended elsewhere.^7^ Studies that aimed at other variables among children and adolescents also did not present data differentiating the age of girls and boys.^10^ These stratifications would give a better understanding of disease mechanisms during childhood and adolescence and help the maintenance of a healthy lifestyle from childhood into adulthood. Thus, the aim of this study was to analyze the associations between sex and age with behaviour related to physical activity practice and sedentary behaviour in children and adolescents.

## Method

**Figure 1:**
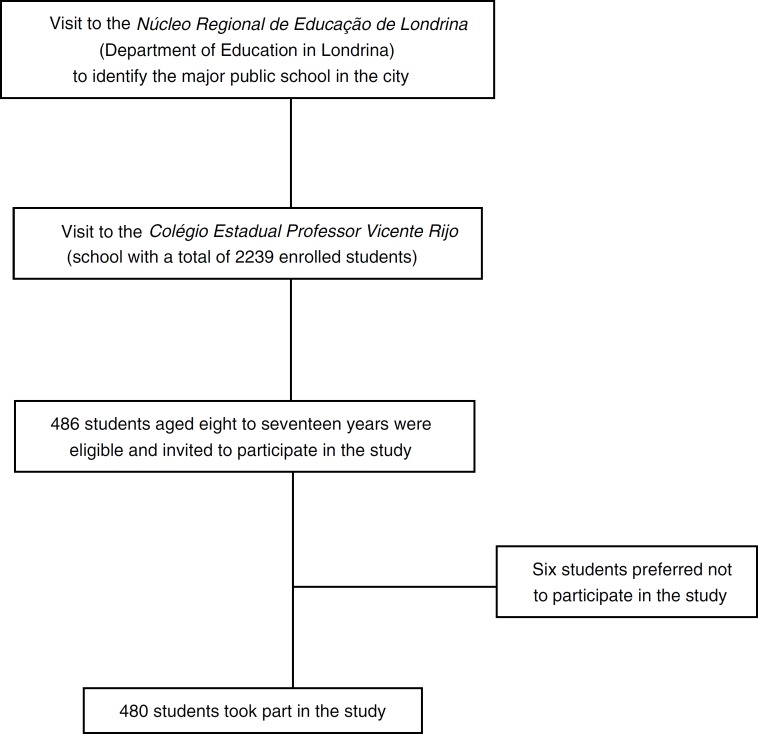
Flow chart explaining the selection process of the sample.

This study had a cross-sectional design. Data collection took place during the second semester of 2011 in the city of Londrina, the fourth largest city in the southern region of Brazil. The city of Londrina has a population size of 543,003 inhabitants, with a Human Development Index of 0.778. It is the second largest city in the state of Parana after the capital, Curitiba. The city has a stable economy and according to its Gross Domestic Product it is ranked as the richest city in the north of Parana.^11^ This study was approved by the Ethics Committee on Research with Human Subjects of the Universidade Estadual de Londrina (CAAE 0089.0.268.000-11) (Fig. 1).

In order to compose a sample of boys and girls aged 8-17 years, the major school in the city was chosen and all students from the 3rd to 8th grades in this school were invited to participate in the study. The school has a total area of 54.000m^2^, is located in the central zone of the city and is the main school in the municipal area. As the school is located in the central zone of the city and it has students from different municipal regions, it was possible to find a large variety of students from different socioeconomic status. The school had a total amount of 2239 students. A total of 486 students enrolled from 3rd to 8th grades, all residents of the city where the study took place. These students were eligible and invited to participate in this study; inclusion criteria for joining the study were: (1) an age of eight to 17 years, (2) students who manifested interest in participating after invitation, and (3) students and parents who returned the questionnaire and the signed consent form with information about the study. No power analyses were completed for the sample size.

The physical activity score was measured using the validated^12^ Physical Activity Questionnaire for Older Children (PAQ-C)^13^ translated into the Portuguese language and adapted by Silva and Malina^14^ to apply to the context of Brazilian students. Thus, no reproducibility assessment of the PAQ-C was made in this study. The students filled out the questionnaire inside their classroom under the supervision of researchers previously trained for its application. The PAQ-C investigates the amount of moderate and intense physical activity carried out in the seven days prior to completing the questionnaire. It is composed of 13 questions on playing sports and games and physical activities at school and during leisure time, including weekends, during the school year. Answers were given on a 5 point Likert-type scale ranging from ‘very sedentary’ to ‘very active’. Scores 2, 3, and 4 represented the categories ‘sedentary’, ‘moderately active’ and ‘active’, respectively. Therefore, from the final score, it was possible to classify the students as physically active or insufficiently active, according to Crocker and Bailey.^13^ Those with scores ≥3 were considered active and those with scores <3 were considered insufficiently active.^13^^,^ 14

Time spent watching television, using the computer and playing videogames use was assessed and defined as screen time.^2^ According to the current recommendations based on self-reports and direct measurements,^2^ a screen time of ≥2h/day was categorized as high sedentary behaviour, whereas a screen time <2h/day was categorized as low sedentary behaviour.

The evaluation of body mass and height of boys and girls was conducted inside the classroom on the same day of the questionnaire's application. The body mass was assessed using a weight scale with a variation range of 0.1-150kg (Britânia, Curitiba, Brazil). Before weight assessment, the subjects removed their shoes and then stood positioned in the centre of the weighing scale platform wearing light clothes. For the height, a stadiometer with a precision range of 0.1cm (Sanny, São Bernardo do Campo, Brazil) was used. After obtaining body mass and height, the body mass index (BMI) using the specific reference values for gender and age proposed by Cole and Lobstein^15^ was calculated. Each subject was classified in accordance with his or her nutritional status: eutrophic, overweight or obese.

After these procedures, students filled out another questionnaire^16^ created by the Brazilian Association of Research Companies for the assessment of the family's economic status. The questionnaire was developed in accordance with the life conditions of Brazilian families. The students’ families were classified into classes: A, B, C, D and E and then divided into high/middle (classes A and B) or low class (classes C, D and E).

The Mann-Whitney *U* test was utilized to compare age variables from both genders and the chi-square test was used for categorical analysis. Poisson regression was used to construct a model for the observed associations. To analyze the degree of the associations between variables, prevalence ratios and confidence intervals of 95% were used. All cases of significance (*p*-value) less than 5% were considered statistically significant. Analyses were performed on the statistical software SPSS (Statistical Package for the Social Sciences Inc., Chicago, Illinois), version 20.0.

## Results

A total of 480 students, consisting of 236 boys and 244 girls aged eight to 17 participated in the study. Six students were not able to join the study as they refused to participate, *i.e.* due to shame of exposing their body weight or body type during the anthropometry measurements or due to the fact that their parents did not return the questionnaires.

Overall, the majority of the sample (boys=62.2%; girls=69.9%), spent more than two hours/day with activities related to screen, *i.e.* television, computer or videogames (PR=1.05 [0.99-1.12]). The prevalence of physical inactivity was also high (boys=80%; girls=91%) in both genders (PR=0.95 [0.92-0.98]). The students’ economic classes found were: A=8.4%, B=67.1%, C=20.2%, D=0.8% and E=34%. Table 1 shows the descriptive analysis according to age, weight, height, BMI and physical activity levels according to the PAQ-C, and sedentary behaviours and comparisons of both genders. According to the PAQ-C score, boys showed higher levels of physical activity when compared to girls (boys=2.4; girls=2.0; *p*<0.001). Girls spent more hours per day with sedentary behaviour than boys (boys=2.4; girls=3.0; *p*=0.026).

**Table 1:** Descriptive analysis of boys and girls.

	Girls				Boys			*p* -value^a^
	P25	Median	P75		P25	Median	P75	
Age (years)	11.7	13.4	14.2		11.7	12.9	14.2	0.430
Weight (kg)	42.1	48.2	56.7		39.0	49.3	58.1	0.983
Height (cm)	150.5	157.0	161.6		147.9	158.2	166.5	0.181
Body mass index	17.6	19.2	22.8		17.0	19.3	22.2	0.262
PAQ-C score	1.6	2.0	2.4		2.0	2.4	2.8	**<0.001**
Sedentary behaviour (h/day)	1.4	3.0	4.3		1.4	2.4	3.7	**0.026**

Body mass index according to Cole and Lobstein (2012). Bold indicates *p*<0.050.

a Mann-Whitney *U* test.

Table 2 shows associations between low levels of physical activity and independent variables in students. High levels of physical inactivity were found in boys aged 8-12 (72.6%), 13-17 years (87.6%; PR=1.09 [1.03-1.15]) and girls aged 8-12 (86.7%) and 13-17 years (94.8%; PR=1.04 [1.00-1.09]). After adjusted analysis, the prevalence of physical inactivity was found to be higher in girls (91%; PR=0.95 [0.92-098]). Boys (87.6%; PR=1.09 [1.03-1.15]) and girls (94.8%; PR=1.04 [1.00-1.09]) aged 13-17 years showed a higher prevalence of physical inactivity than younger peers. Table 3 shows associations between high screen time and independent variables in students. When analyzing older boys and girls together, a higher prevalence of high screen time than their younger peers was found (71.8%; PR=0.91 [0.85-0.97]). When comparing older boys to younger boys, the prevalence of older boys with high screen time was higher than in younger boys (69.7%; PR=0.90 [0.82-0.98]).

**Table 2:** Association between low levels of physical activity and independent variables in children and adolescents.

	Inactive		
	*n*=409 (85.2%)	PR (95%CI)^a^	PR (95%CI)^b^
*Sex*			
Male	196 (80.0)	0.95 (0.83-1.09)	0.95 (0.92-0.98)^c^
Female	213 (91.0)		

*Age (both genders)*			
13-17	234 (91.4)	1.06 (0.92-1.21)	1.06 (1.02-1.10)^c^
8-12	174 (78.7)		

*Male*			
13-17	106 (87.6)	1.09 (0.90-1.31)	1.09 (1.03-1.15)^c^
8-12	90 (72.6)		

*Female*			
13-17	128 (94.8)	1.04 (0.86-1.26)	1.04 (1.00-1.09)^c^
8-12	85 (86.7)		

*Economic status*			
High/middle	132 (81.0)	1.03 (0.89-1.19)	1.03 (0.99-1.07)
Low	259 (87.5)		

*Screen time*			
Less than two hours/day	129 (81.1)	0.97 (0.84-1.12)	0.97 (0.93-1.01)
Two or more hours/day	269 (87.9)		

*Body mass index*			
Eutrophic	290 (85.5)	1.01 (0.87-1.18)	1.01 (0.97-1.05)
Overweight	88 (86.3)		
Obese	31 (85.6)		

a Crude analysis.

b Analysis adjusted by all variables, independently of *p*-value from crude analysis.

c *p*<0.050.

**Table 3:** Association between high screen time and independent variables in children and adolescents.

	Screen time≥2h/day		
	*n*=306 (63.8%)	PR (95%CI)^a^	PR (95%CI)^b^
*Sex*			
Male	148 (62.2)	1.05 (0.89-1.24)	1.05 (0.99-1.12)
Female	158 (69.6)		

*Age (both genders)*			
13-17	181 (71.8)	0.91 (0.77-1.07)	0.91 (0.85-0.97)^c^
8-12	124 (58.5)		

*Male*			
13-17	83 (69.7)	0.90 (0.72-1.11)	0.90 (0.82-0.98)^c^
8-12	65 (54.6)		

*Female*			
13-17	98 (73.7)	0.93 (0.74-1.17)	0.93 (0.85-1.02)
8-12	60 (63.8)		

*Economic status*			
High/middle	113 (69.3)	1.05 (0.88-1.24)	1.05 (0.98-1.12)
Low	193(65.6)		
*Physical activity*			
Active	37 (55.2)	0.93 (0.75-1.17)	0.93 (0.85-1.02)
Inactive	269 (67.6)		

*Body mass index*			
Eutrophic	215 (65.3)	1.00 (0.84-1.20)	1.00 (0.93-1.08)
Overweight	68 (67.3)		
Obese	23 (65.8)		

a Crude analysis.

b Analysis adjusted by all variables, independently of *p*-value from crude analysis.

c *p*<0.050.

## Discussion

The aim of this study was to analyze the associations between sex and age with behaviour related to physical activity practice and sedentary behaviour in children and adolescents. Comparing different gender groups in childhood and adolescence, girls showed lower physical activity levels than boys. The results from this study support previous findings. Decelis et al.^17^ reported that a high percentage of boys and girls are not meeting physical activity recommendations^1^ and show that levels of physical activity in childhood and adolescence start decreasing before adulthood. Family plays an important role in physical activity practice in childhood and adolescence.^18^ One explanation for boys engaging in more physical activity than girls is that they seem to have more social and family support for practicing physical activity.^19^ There is still a need to promote physical activity in childhood and adolescence and this data can help to develop interventions for this population. These comparisons deliver information to the literature as recommended before for further studies.^7^

Comparisons made with girls from different age groups showed that older girls spend more screen time than younger girls. Consequences of high amounts of time spent with sedentary activities are expected in early childhood. A study of physical activity and obesity trends reported by Sigmundová et al.^7^ showed that, over a period of ten years, the time spent with sedentary activities increased and the level of physical activity decreased in childhood and adolescence. Cluster analysis conducted by De Bourdeaudhuij et al.^20^ with children recruited from Hungary, Belgium, the Netherlands, Greece and Switzerland showed that girls spent more time being sedentary than boys, similar to our findings. Sedentary activities of boys and girls are higher than the current recommendations,^2^ and programmes focusing on both decreasing sedentary behaviour and increasing physical activity are needed, particularly in girls.^21^ Lower levels of physical activity among older boys and girls might be explained by the fact that parents can associate lower academic achievement at school with the time that they spend outside the home, which might be a barrier for older boys and girls to engage in more physical activity.^19^

In this study, we found a higher prevalence of older male students spending more screen time and practicing less physical activity than younger boys. An explanation for this difference found in our study could be that many older boys have attributes that children still do not have, *i.e.* job or study obligations.^22^ These types of routines are common between middle-class male and female adolescents in Brazil.^22^ However, our study did not include specific information about daily tasks out of school besides physical activity and sedentary behaviour.

The prevalence of sedentary behaviour found in the present study was high in both genders, and this corroborates recent findings of a Brazilian study by Silva et al.,^23^ where the authors investigated the association between sports participation and sedentary behaviour and found that the majority of the adolescents included in their sample had a high incidence of sedentary behaviour. Suchert et al.^24^ assessed effects of sedentary behaviour, depressed affect, self-esteem, physical self-concept, general self-efficacy and physical activity. Among girls, lower scores in self-esteem and general self-efficacy were associated with higher screen-based sedentary behaviours. Melkevik et al.^25^ reported that the use of electronic media was associated with increased BMI *z*-scores and higher odds of being overweight in boys and girls who did not follow the physical activity guidelines. Recent research^23^ found a negative association between sedentary behaviour and engagement in sports in adolescents.

A high prevalence of physical inactivity was found in students with high screen time. Several studies analyzed these co-existent variables in this population. Physical activity and more time spent with sedentary behaviour are related to academic skills.^26^ Additionally, low levels of physical activity and high levels of sedentary behaviours increase the chances of obesity in childhood.^1^^,^ 2 Obesity in childhood and adolescence is linked to numerous chronic diseases in life. A Brazilian study conducted by Dutra et al.^27^ reported a prevalence of sedentary lifestyle of more than 70% and that screen time was inversely associated with physical activity. Similarly, Ferrari et al.^28^ found a higher prevalence of children meeting moderate to vigorous physical activity guidelines among children who watched ≤2h/day of television. Still, insufficient physical activity should not be related to sedentary behaviours as it is not directly linked to sedentary activities investigated in this study, *i.e.* television viewing.^29^ Evidence shows that television viewing and physical activity in childhood and adolescence are non-related constructs,^29^ and practicing more physical activity does not necessarily lower sedentary behaviours.^30^ Our findings raise major concerns and they corroborate the high prevalence of both risk factors, high sedentary behaviour and low physical activity, reported elsewhere, in different regions of Brazil.^23^^,^ 28

This study has limitations which must be taken into account: first, the method of investigation relies on self-reported questionnaires regarding physical activity and sedentary behaviours. There are advantages using these methods, *i.e.* full description and details about the physical activity and time spent with sedentary behaviour; however motion sensor devices would deliver better and more precise information compared to the 7 day recall method. Secondly, the cross-sectional design prevents the assessment of causality. Longitudinal design might allow better understanding, instead of making comparisons between younger and older students from different regions of the town and different social classes. The sample used in this study is not representative of all students of the city; however it is representative of the major school in the city where the study took place. Additionally, the selected school for this study has students from all regions of the city. However, data collection from other cities would give a larger sample size, and make comparisons between similar studies from other countries possible.^7^

In conclusion, our results support evidence that physical activity levels are lower in older students than younger students. Additionally, older students spent more time with sedentary activities than their younger peers. The prevalence of physical inactivity was higher in girls than in boys. Older boys showed lower levels of physical activity and higher amounts of screen time than younger boys.
